# Physical activity and academic achievement among Norwegian adolescents: Findings from a longitudinal study

**DOI:** 10.1016/j.pmedr.2021.101312

**Published:** 2021-01-09

**Authors:** Ingeborg Barth Vedøy, Knut Ragnvald Skulberg, Sigmund Alfred Anderssen, Hege Eikeland Tjomsland, Miranda Thurston

**Affiliations:** a Inland Norway University of Applied Sciences, Postboks 400, 2418 Elverum, Norway; b The Norwegian School of Sport Sciences, Postboks 4014 Ullevål stadion, 0806 Oslo, Norway

**Keywords:** Adolescence, Accelerometry, Academic performance, Sleep, Body composition

## Abstract

Regular physical activity (PA) has been associated with academic achievement, but the evidence is mainly based on cross-sectional research and self-reported measures of PA. The purpose of the current study was to explore the longitudinal relationship between objectively measured PA and academic achievement among a cohort of adolescents in Norway between 2016 and 2018. As a secondary aim, an indirect relationship via waist circumference (WC) and sleep duration was assessed. Data from 599 adolescents (54.4% female, mean age at baseline ± SD 13.3 ± 0.3 years) were collected annually during their three years at lower secondary school. PA was measured objectively using accelerometry. Academic achievement was assessed using grade point average (GPA) from school records. Linear regression analysis was performed to explore associations between longitudinal changes in measures of PA (Total PA and moderate-to-vigorous PA [MVPA]) and academic achievement directly or via mediators. Results showed no significant associations between Total PA or MVPA and academic achievement, either in the main analyses or through mediation of WC and sleep duration. The results contribute to a growing evidence base of studies showing no association between objectively measured PA and academic achievement among adolescents.

## Highlights

• A longitudinal study of youth explored physical activity and academic achievement.
• Physical activity was assessed by accelerometry and academic achievement by school grades.
• Mediation through changes in waist circumference and sleep duration were explored.
• Changes in physical activity were not associated with changes in academic achievement.
• There was no mediation via changes in waist circumference or sleep duration.

## 1 Introduction

Succeeding academically during compulsory education is associated with broad and enduring advantages for later educational success, income, and better physical and mental health (Marmot, 2010). This well-documented association highlights the importance of identifying factors that might contribute to academic achievement. There has long been an assumption that regular physical activity (PA) could positively contribute to academic achievement via enhancement of cognition (Lubans et al., 2016).

The volume of studies exploring the association between PA and academic achievement has increased substantially in recent years, alongside an increase in systematic reviews (Donnelly et al., 2016, Marques et al., 2017, Poitras et al., 2016, Singh et al., 2018). However, results are inconsistent, and most reviews conclude that there is a need for further research that addresses the methodological weaknesses of studies. The majority of studies have used a cross-sectional design, which is subject to cohort effects and cannot provide information about changes in predictor or outcome experienced by a single individual (Dumith et al., 2011). A longitudinal design can overcome these inherent limitations and is thus preferable when exploring associations between predictor and outcome variables that might develop over time.

The lack of comparable measurements across studies has been put forward as an additional explanation for the inconsistency in findings (Marques et al., 2017). Studies in which PA has been assessed by self-report tend to find an association with academic achievement (Kantomaa et al., 2016, Kristjansson et al., 2010, Suchert et al., 2016), whereas those in which PA has been objectively measured frequently report null-findings (Corder et al., 2015, Oliveira et al., 2017, Syväoja et al., 2013, van Dijk et al., 2014). However, the number of studies that have used an objective measurement of PA remains small (Owen et al., 2018). In their review, Marques et al. (2017) explored the relationship between academic achievement and PA both by self-report and objective measures. They found that while self-reported PA was consistently and positively associated with academic achievement, there was an inconsistent relationship between objectively measured PA and academic achievement. There was, nevertheless, nothing to suggest that there was a detrimental effect of PA. This is also supported by Syväoja et al. (2013) who assessed intensity-specific PA both by accelerometer and self-report. They found no association between objectively measured moderate-to-vigorous physical activity (MVPA) and grade point average (GPA), whereas they found a positive association between self-reported MVPA and GPA. They suggest that this inconsistency might be explained by these measurements not necessarily measuring the same construct of PA. Children and adolescents are especially at risk of over- or underestimating PA levels through self-report because of a more pronounced tendency than adults for recall and social desirability biases (Adamo et al., 2009). Marques et al. (2017) thus state that conclusions based solely on self-reported PA should be interpreted with caution. Given that objective measures can produce more robust estimates of PA (Adamo et al., 2009), their use has been advocated (Poitras et al., 2016).

PA is often quantified as total volume (total PA) or time spent in different intensities (Chomistek et al., 2017). Previous search shows that there might be differences in relationship with academic achievement depending on PA measure used (Booth et al., 2014), where positive associations have been reported more frequently for higher intensities (Kwak et al., 2009). However, research also shows evidence of a negative relationship between MVPA and academic achievement (Esteban-Cornejo et al., 2014, Estrada-Tenorio et al., 2020). There is, therefore, a need to explore this further.

Any association between PA and academic achievement might not, however, be direct, but indirect and act via mediators. Evidence regarding potential biological or psychosocial mediators is scarce, indicating a need for mediation studies (Singh et al., 2018). Obesity has been shown to be negatively related to both PA and cognitive functioning (Chang et al., 2017) and its role in the hypothesised relationship between PA and cognition has been explored in a review by Chang et al. (2017). They found two studies exploring weight status as a potential mediator of the association between PA and cognition, both conducted on an adult population. Although both studies found evidence of a mediating effect of obesity, only one study was reported to sufficiently test the mediator model. Evidence of mediation among adults was therefore limited. However, Lima et al. (2019) found an association between both MVPA and VPA and academic achievement mediated by body composition measured by waist circumference (WC) among children. To our knowledge, this has not previously been explored among adolescents.

Lubans et al., (2016) propose that changes in cognitive functions resulting from PA might be mediated by changes in associated behaviours, such as sleep duration. Khan et al. (2015) found that longer sleep duration was associated with higher levels of PA, and in a systematic review (Chaput et al., 2016), evidence of an association between longer sleep duration and better academic achievement among children and youth was found. Sleep duration might then serve as a mediator in the relationship between PA and academic achievement and has not, to our knowledge, been explored hitherto. Syväoja et al. (2018) found a negative indirect association between self-reported MVPA and academic achievement via later bedtime, solely among girls. However, no relationship was present for objectively measured MVPA.

The purpose of this study is, therefore, to explore whether longitudinal changes in objectively measured PA [Total PA and MVPA] are associated with changes in academic achievement directly or via mediation of WC or sleep duration. We also explore whether there are differences between boys and girls.

## 2 Method

### 2.1 Design and participants

A prospective cohort study was carried out, which collected annual data from adolescents in 11 different schools during their three years in lower secondary school (normal age range 12–16 years). The schools were purposively recruited from three counties on the east and west side of Norway and were selected on the basis of school size, type of school (grades 1–10 or 8–10) location (urban, suburban and rural), socio-economic status (SES) and schools average score on National tests. Detailed description of the methodology has been published elsewhere (Barth Vedøy et al., 2020).

**Fig. 1:**
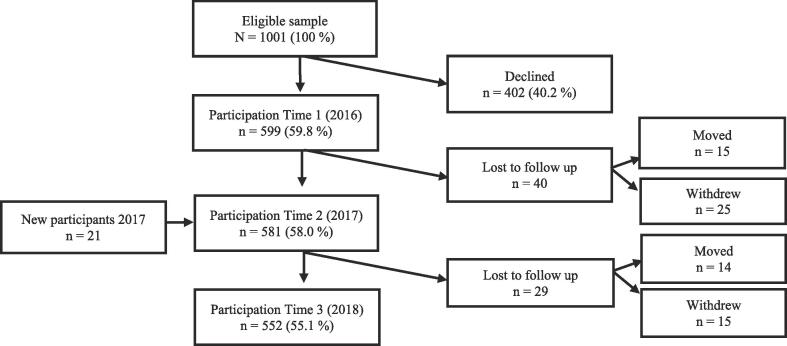
Study population and participation.

Data collection was conducted annually during the first semester of 8th, 9th, and 10th grade (2016–2018) for the predictor and mediator variables and at the end of first semester for the outcome variable. The study population consisted of all consenting adolescents starting in 8th grade in the autumn of 2016. Fig. 1 shows the number of adolescents invited and the proportion who participated throughout the three waves of the study.

### 2.2 Measurements

#### 2.2.1 Physical activity

Physical activity was measured objectively using accelerometry (ActiGraph GT3X+ and GT3X-bt, LLC, Pensacola, Florida, USA). The monitor was placed on the right hip in an elastic belt. The participants were asked to wear it for seven consecutive days. Research staff attached the monitor during the first school visit to ensure correct placement. The participants were instructed to wear the monitor during all waking hours except while swimming or doing other water activities. The monitor was initialised to start recording at 06:00 the day after it was delivered to the participants. After excluding data recorded between 00:00 and 06:00 and all intervals of ≥20 consecutive minutes of no recording (non-wear), data were included if the participants had ≥480 min of valid activity recordings each day. Analysis showed that participants with 2–3 days (Time 1 and 2 [T1 and T2]) and 1–3 days (Time 3 [T3]) of valid measurements did not differ from those with ≥4 days, and were thus included in further analysis. A 10-second epoch was used to register number of counts. For intensity specific analyses, data were aggregated to a 60-second epoch. The use of these criteria are in accordance with previous research using ActiGraph on similar age groups (Dalene et al., 2018b, Owen et al., 2018). As an overall measure of PA (Total PA), average counts·min^−1^ (CPM) throughout the assessment period was used. We defined sedentary behaviour (SED), light PA (LPA) and moderate to vigorous PA (MVPA) as <100 CPM, 100-1999 CPM and ≥2000 CPM respectively. These cut-points are in accordance with previous research on adolescents in Norway (Dalene et al., 2018b).

When returning the accelerometer, participants were asked to register the amount of time spent doing activities that are poorly measured with the accelerometer (cycling) or required removal of the device (swimming). Time spent swimming and/or cycling among the majority of participants in this study was very low and these data were thus not included in further analyses.

#### 2.2.2 Academic achievement

Academic achievement was assessed through the midterm grades from school records. Grade point average (GPA) was used as the indicator of academic achievement. GPA represents the average score achieved by the pupil in all subjects. In Norway, pupils in the same grade follow the same core subjects. The grade range is 1–6 where 6 represents the highest achievement possible. In the post-hoc analysis, the study sample was divided into a low GPA group (GPA 1.00–3.99) and a high GPA group (GPA 4.00–6.00) based on GPA T1.

#### 2.2.3 Waist circumference

WC was measured to the nearest 0.5 cm with a medical measuring tape midway between the lowest rib and the top of the iliac crest at the end of gentle expiration. Measurements were conducted twice if the difference was ≤1 cm and three times if the difference was >1 cm. The average value of the two closest measurements was used.

#### 2.2.4 Sleep

The participants reported the average times for going to bed and getting up in the morning on school days in a questionnaire (Supplementary Table S1). To estimate sleep duration, we subtracted and added 30 min to the lower (before 06:30/20:00) and upper (after 08:00/24:00) categories, respectively. In the remaining categories, the midpoint was used (e.g. 07:00–07:30 recoded to 07:15). The following algorithm was used for participants going to bed before 24.00: (sleep duration = 24 – CTIME.HOURS (bedtime – out of bed)) and (sleep duration = CTIME.HOURS (out of bed – bedtime)) for those going to bed after 24.00. This yielded sleep duration in hours for the participants (Dalene et al., 2018a).

#### 2.2.5 Additional covariates

According to previous research, the following variables may influence the variables of interest: sex, body mass index (BMI), SES and seasonality (Faught et al., 2017, Owen et al., 2018, Rich et al., 2012). BMI was calculated using weight and height (kg·m^−2^). Weight and height were measured to the nearest 0.1 kg (Seca 877, SECA GmbH, Hamburg, Germany) and 0.5 cm (wall-mounted measuring tape), respectively. Participants were asked to remove shoes and sweaters. In line with standard practice, bodyweight measures were adjusted by subtracting 0.3 kg to account for clothing (Dalene et al., 2018b).

SES was measured through The Family Affluence Scale. This scale measures material affluence, and can be used as a proxy for SES (Hartley et al., 2016). Based on this scale, a score of *relative family affluence* was constructed by summing scores on all answers and categorising them in three broader groups (the lowest 20%, the middle 60% and the highest 20%).

Seasonality was defined by two categories depending on the time of data collection at the different schools (1: Sept–Oct, 2: Nov–Jan).

### 2.3 Data analyses

**Fig. 2:**
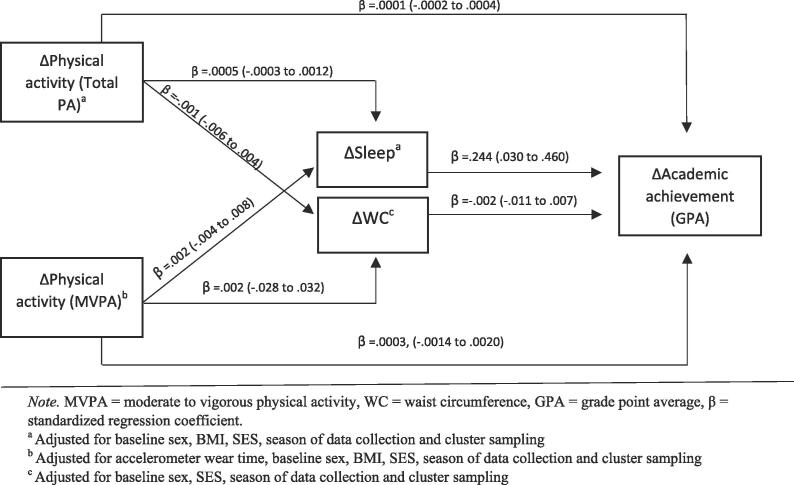
Parameter estimates of the regression coefficient of the longitudinal associations between changes (2016–2018) in physical activity (Total PA and MVPA), WC, sleep duration and academic achievement. (n = 379–468).

Statistical analyses were performed using IBM SPSS Statistics for Windows, Version 24.0 and Stata Statistical Software, version 16.0 (Copyright 1985–2019 StataCorp LLC), Texas 77,845 USA. Descriptive data are presented as frequencies, mean and SD, and 95% confidence intervals (CI) where appropriate. Hayes’ Process Macro v3.5 for SPSS was used to explore interactions by sex. As analyses showed no interaction, all data were analysed collectively. To test whether changes in objectively measured PA [Total PA and MVPA] was associated with changes in academic achievement directly or via mediators, variables of change (Δ) were created by subtracting T3-values from T1-values. Associations between the change variables were analysed by linear regression, adjusted for T1 covariates including sex, BMI, SES and seasonality (Fig. 2). All intensity specific analyses were adjusted for wear time of the accelerometer. Mediation of WC or sleep duration was explored through pairwise analyses between predictor and outcome, predictor and hypothesised mediators, and hypothesised mediators and outcome. Further, post-hoc analysis of groups with low GPA (1.00–3.99) and high GPA (4.00–6.00) were conducted to explore whether the pattern differed between these groups.

To test whether changes in PA [Total PA and MVPA] prospectively could indicate academic achievement at T3, linear regression analyses were used, adjusted for T1 covariates. Finally, linear regression analyses of each time point were conducted in order to provide a descriptive supplement to the longitudinal analyses (Table 2). The crude model showed associations between Total PA and academic achievement, whereas the adjusted model corrected for sex, BMI, SES and seasonality. All measures were scaled up to show changes in dependent variables occurring after changes in Total PA of 100 CPM. For informational purposes, separate analyses for boys and girls are presented in Supplementary file, Table S2.

Because of the clustered nature of the data, all analyses were adjusted for school-level clustering.

### 2.4 Ethics

The study was approved by the Norwegian Centre for Research Data (project no. 48192). As the participants were between 12 and 13 years at the time of enrolment in the study, written informed consent was obtained from pupils and his/her legal guardian prior to data collection.

## 3 Results

### 3.1 Descriptive results

BMI, WC and GPA significantly increased in both boys and girls from T1 to T3, while amount of sleep decreased significantly. The two measures of PA decreased significantly among girls from T1 to T3 (p = .020 for Total PA and p ≤ 0.001 for MVPA). This decrease was not found among boys (p = .589 for Total PA and p = .085 for MVPA) (see table 1).

**Table 1:** Descriptive characteristics of the study sample by sex, all time points (mean ± SD unless otherwise specified).

Characteristic	Time 1 (2016) n = 599	Time 2 (2017) n = 581	Time 3 (2018) n = 552
Age			
Boys	13.4 (0.3)	14.4 (0.3)	15.4 (0.3)
Girls	13.3 (0.3)	14.3 (0.3)	15.3 (0.3)
Height (cm)			
Boys	163.9 (8.6)	171.3 (8.2)	176.9 (7.5)
Girls	161.7 (6.9)	165.0 (6.6)	166.3 (6.4)
Weight (kg)			
Boys	53.0 (11.8)	60.7 (13.5)	66.8 (13.6)
Girls	52.8 (9.9)	56.6 (9.8)	59.2 (9.8)
BMI (kg/m^2^)			
Boys	19.6 (3.3)	20.5 (3.7)	21.3 (3.7)
Girls	20.0 (3.1)	20.8 (3.0)	21.4 (3.0)
WC (cm)			
Boys	73.2 (9.7)	75.3 (10.0)	76.4 (9.4)
Girls	70.5 (8.8)	71.4 (7.7)	70.9 (7.4)
Sleep duration (h)			
Boys	9.3 (0.9)	8.7 (0.9)	8.4 (0.9)
Girls	9.1 (0.8)	8.7 (0.9)	8.4 (0.9)
Total PA (CPM/d)			
Boys	474.4 (178.7)	476.8 (177.5)	460.9 (204.7)
Girls	402.2 (118.6)	399.9 (127.5)	383.5 (138.3)
SED (min/d)			
Boys	548.4 (74.4)	541.7 (78.7)	542.1 (99.6)
Girls	565.7 (55.4)	561.3 (64.6)	564.1 (69.6)
LPA (min/d)			
Boys	172.2 (34.1)	167.6 (34.3)	140.8 (37.3)
Girls	161.4 (32.3)	153.9 (32.2)	137.6 (30.4)
MVPA (min/d)			
Boys	63.9 (22.9)	62.4 (24.3)	58.1 (26.7)
Girls	54.0 (18.5)	53.6 (19.4)	50.1 (21.4)
Meeting act. reg (%)			
Boys	53.3	53.6	43.8
Girls	36.3	37.1	29.5
GPA			
Boys	3.8 (0.6)	3.9 (0.7)	4.0 (0.7)
Girls	4.1 (0.7)	4.3 (0.7)	4.4 (0.8)

*Note.* BMI = body mass index, WC = Waist circumference, Total PA (CPM) = average daily counts per minute, SED = average daily sedentary behaviour, LPA = average daily light physical activity, MVPA = average daily moderate to vigorous physical activity, Meeting act.reg = Meeting national recommendations of an average of ≥60 min MVPA per day, GPA = grade point average, CPM/d = counts per minute per day, min/d = minutes per day.

**Table 2:** Cross-sectional associations between Total PA and academic achievement analysed with a multiple linear regression model (n = 402–570).

	Crude^a^				Adjusted^b^			
	n	β	95% CI	p	n	β	95% CI	p
GPA Time 1	570	−0.009	−0.053, 0.035	0.650	518	0.006	−0.041, 0.054	0.767
GPA Time 2	499	0.026	−0.010, 0.061	0.137	435	0.045	0.006, 0.084	0.028
GPA Time 3	458	−0.021	−0.061, 0.020	0.289	402	−0.010	−0.062, 0.041	0.670

*Note.* GPA = grade point average, β = standardized regression coefficient.

All measures are scaled up 100 times showing changes in dependent variables occurring after changes of 100 CPM.

a Adjusted for cluster sampling.

b Adjusted for cluster sampling, sex, BMI, SES and season of data collection.

### 3.2 Longitudinal results

Neither change in Total PA nor MVPA were directly associated with change in academic achievement (see Fig. 2). No indirect associations via change in WC or sleep duration were present. Post-hoc analyses, where the study sample was divided into high and low GPA groups were conducted (data not shown). The results from these analyses did not deviate from the results of the main analysis.

**Fig. A1:**
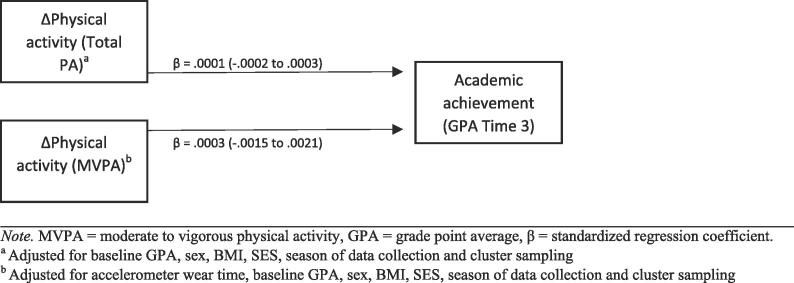
Parameter estimates of the regression coefficient (95% CI) of the prospective associations between changes (2016–2018) in physical activity (Total PA and MVPA) and academic achievement Time 3. (n = 401).

Results from the prospective analyses showed no associations between change in Total PA or MVPA and academic achievement at T3 (see Fig. A1).

### 3.3 Cross-sectional results

Table 2 shows the cross-sectional associations between Total PA and academic achievement measured by GPA at T1, T2 and T3. Total PA was significantly associated with academic achievement in T2, but solely in the adjusted analysis (p = 0.028) and the association was weak. Gender specific analyses (Supplementary Table S2) shows that this association was only significant among boys.

## 4 Discussion

The aim of the study was to explore whether the longitudinal change in objectively measured PA [Total PA and MVPA] was associated with change in academic achievement directly or via mediators. We found no association between longitudinal changes in PA levels [Total PA and MVPA] and academic achievement in the study sample of Norwegian adolescents, not by analysing the study sample as a whole, by grade specific analysis, or through mediators. There are several possible explanations for these findings.

Previous research shows a diverse picture of the association between PA and academic achievement, and results seem to be somewhat dependent on the way PA is measured. The current study adds to this by showing no significant association between objectively measured PA and academic achievement in the longitudinal analyses. Although assessing PA by an objective measure generates information about duration of movements with different intensities and is generally regarded as producing more robust estimates (Adamo et al., 2009), it does not indicate type of activity (Marques et al., 2017, Syväoja et al., 2013). Given the possible relationship between motor control and cognitive development, skill-specific activities that do not generate activity counts might be relevant in explaining the relationship between PA and academic achievement (Marques et al., 2017), an issue that might be addressed in future research. However, analysis showed a cross-sectional association between Total PA and academic achievement at T2. It is possible that the association between PA and academic achievement differs between the years at lower secondary school, although the association was very small, and its consequence in real life difficult to infer. Furthermore, a longitudinal design generates more robust data than a cross-sectional design, putting a stronger emphasis on the results from the former.

Longitudinal associations were explored via change variables of both predictor and outcome. This might affect the results in situations where stability of variables over the follow-up period is high. In the current study, PA decreased significantly among girls, and GPA increased significantly among both boys and girls from T1 to T3. Stability of data is therefore unlikely to explain the results. A ceiling effect might also be an explanation for lack of association when change scores are analysed (Resaland et al., 2016). Those with already high GPA from T1 do not have the same possibility for further increase as those with lower GPA. Post-hoc analyses explored this potential explanation and found no evidence to suggest that a ceiling effect could explain the lack of significant associations in the current study sample.

Adolescence is an extended period of considerable physical and cognitive development (Paus, 2005). The follow-up period may not have been sufficiently long for associations between PA and academic achievement to emerge. A longitudinal study based on self-reported PA with a 30-year follow-up found long-term associations between change in PA (between the years of 12 and 15) and subsequent educational attainment in adulthood (Kari et al., 2017). However, when adjustments for individuals’ prior academic achievement were included, the strength of the associations decreased. This suggests that the results are partly influenced by the higher GPA of physically active adolescents, and further raises the question about a selection effect.

In contrast to Lima et al. (2019), the current study showed no mediation effect via WC, neither through analysis of the entire study sample, or grouped by low- and high GPA. While the current study used GPA as a measure of academic achievement, Lima et al. (2019) used a composite score of national standardised tests in Danish and Mathematics. School grades assess a broad range of competencies, but might be influenced by teachers’ perceptions (Marques et al., 2017, Owen et al., 2018). Although standardised tests yield an unbiased measure, they only assess a narrow range of the skills and competencies of the pupil (Owen et al., 2018, The Norwegian Directorate for Education and Training, 2019). These differences in outcome measure might serve as an explanation for the dissimilar results. In addition, Lima et al. (2019) targeted a younger age group (7–12 years) than the current study (12–16 years). At a theoretical level, associations might be present among children without tracking into adolescence.

Neither the main nor post-hoc analysis showed any evidence for sleep duration serving as a mediator between PA and academic achievement. Although these results contrast with those of Syväoja et al. (2018) where a subjective measure of PA was used, they are consistent with results from the same study where an objective measure was used.

There were no differences between analyses using Total PA and MVPA as predictor variables in the present study, giving no evidence for an intensity-dependent association. This is consistent with previous research which found no direct association between PA and academic achievement in overall analysis of MVPA and/or VPA (Corder et al., 2015, Syväoja et al., 2013, van Dijk et al., 2014). However, Kwak et al. (2009) found a positive association between VPA and academic achievement, solely among girls. In a longitudinal study by Owen et al. (2018), positive associations were found through analysis of associations between changes in MVPA and changes in academic achievement among girls. The enduring differences between studies prevent us from drawing any conclusions regarding the putative role of intensity and gender differences in the analysis of PA and academic achievement. Further research is needed to explore this relationship.

### 4.1 Strengths and limitations

A key strength of the current study is its longitudinal design where both predictor and outcome are measured at all three time points. Even though the initial participation rate was 60%, and may have introduced some selection bias into the results, the attrition rate from T1 to T3 was very low (see Fig. 1). In addition, the analyses controlled for many well-known confounders. The use of objectively measured PA also strengthens the study’s quality. However, use of accelerometers also have some limitations. While worn at the hip and not completely waterproof, their ability to measure upper body movements, load-carrying activities, cycling and swimming are limited (Shephard and Aoyagi, 2012). Time spent swimming and/or cycling among the majority of participants in this study was very low. Consequently, it is unlikely to have led to a large underestimation of activity level. While there remains no consensus regarding cut-points for intensity-specific PA, the present study used the same cut-points as other studies of Norwegian adolescents have done. This may limit comparability across studies using different cut-points. Finally, because the selection of participating schools was not conducted at random, selection bias cannot be ruled out, and generalisability is limited.

## 5 Conclusion

PA was unrelated to academic achievement in the current study sample, both through a direct association, and via mediators. These results contribute to a growing evidence base showing no relation between objectively measured PA and academic achievement among adolescents. However, it is important to note that although no positive association was found, there was nothing to suggest a detrimental effect of PA either, further implying that PA can be promoted for its well-known beneficial effects on health among adolescents. Future research should explore these associations on a long-term basis where important covariates of SES is also accounted for. Finally, there is a need for research to further explore the potential long-term influence of sleep duration on these associations.

## CRediT authorship contribution statement

**Ingeborg Barth Vedøy:** Conceptualization, Methodology, Formal analysis, Investigation, Writing - original draft, Visualization. **Knut Ragnvald Skulberg:** Methodology, Formal analysis, Writing - review & editing, Visualization. **Sigmund Alfred Anderssen:** Methodology, Formal analysis, Writing - review & editing. **Hege Eikeland Tjomsland:** Conceptualization, Writing - review & editing. **Miranda Thurston:** Conceptualization, Project administration, Funding acquisition, Writing - review & editing.

## Declaration of Competing Interest

The authors declare that they have no known competing financial interests or personal relationships that could have appeared to influence the work reported in this paper.

## Appendix A

Fig. A1.
