# Supplementary material for: Physical activity and academic achievement among Norwegian adolescents: Findings from a longitudinal study
Source: Prev Med Rep. 2021 Jan 9;21:101312. doi: 10.1016/j.pmedr.2021.101312 (PMC7841350; doi:10.1016/j.pmedr.2021.101312)
Supplement: Supplementary Data 1 [file mmc1.docx]

**Supplementary file**

**Table S1.** Questions about usual time for going to bed and getting up in the morning on school days from the questionnaire.

| Sleep duration |  |
| --- | --- |
| When do you usually get out of bed on schooldays? | □ Before 06:30, □ Between 06:30 and 07:00, □ Between 07:00 and 07:30, □ Between 07:30 and 08:00, □ After 08:00 |
| When do you usually go to bed on schooldays? | □ Before 20:00, □ Between 20:00 and 21:00, □ Between 21:00 and 22:00, □ Between 22:00 and 23:00, □ Between 23:00 and 24:00, □ After 24:00 |

**Table S2.** Cross-sectional associations between Total PA and academic achievement analysed with a multiple linear regression model (n= 402-570)

|  | **Crude^a^** | | | |  | **Adjusted^b^** | | | |
| --- | --- | --- | --- | --- | --- | --- | --- | --- | --- |
|  | **n** | **β** | **95 % CI** | **p** |  | **n** | **β** | **95 % CI** | **p** |
| **GPA Time 1**  Boys  Girls | 570  254  316 | -0.002  0.05 | -0.05, 0.04  -0.05, 0.15 | .931  .280 |  | 518  237  281 | 0.0002  0.01 | -0.05, 0.05  -0.08, 0.11 | .994  .759 |
| **GPA Time 2**  Boys  Girls | 499  208  291 | 0.05  0.07 | -0.01, 0.11  -0.04, 0.17 | .091  .189 |  | 435  186  249 | 0.04  0.06 | 0.006, 0.07  -0.04, 0.17 | .024  .203 |
| **GPA Time 3**  Boys  Girls | 458  190  268 | -0.01  0.03 | -0.07, 0.05  -0.06, 0.11 | .667  .492 |  | 402  170  232 | -0.02  0.001 | -0.07, 0.03  -0.09, 0.09 | .341  .979 |

*Note.* GPA = grade point average, β = standardized regression coefficient
^a^Adjusted for cluster sampling
^b^Adjusted for cluster sampling, BMI, SES and season of data collection.
All measures are scaled up 100 times showing changes in dependent variables occurring after changes of 100 CPM.
